# Inhibition of Six1 affects tumour invasion and the expression of cancer stem cell markers in pancreatic cancer

**DOI:** 10.1186/s12885-017-3225-5

**Published:** 2017-04-07

**Authors:** Tristan Lerbs, Savita Bisht, Sebastian Schölch, Mathieu Pecqueux, Glen Kristiansen, Martin Schneider, Bianca T. Hofmann, Thilo Welsch, Christoph Reissfelder, Nuh N. Rahbari, Johannes Fritzmann, Peter Brossart, Jürgen Weitz, Georg Feldmann, Christoph Kahlert

**Affiliations:** 1 Department of General, Visceral and Transplantation Surgery, Im Neuenheimer Feld 110, 69120 Heidelberg, Germany; 2 Department of Internal Medicine 3, Center of Integrated Oncology (CIO) Cologne-Bonn, University Hospital of Bonn, Bonn, Germany; 3 Department of Gastrointestinal, Thoracic and Vascular Surgery, Medizinische Fakultät Carl Gustav Carus, Technische Universität Dresden, Fetscherstr. 74, 01307 Dresden, Germany; 4 Department of Pathology, Center of Integrated Oncology Cologne-Bonn, University Hospital of Bonn, Bonn, Germany; 5 Department of General, Visceral and Thoracic Surgery, University Medical Center Hamburg-Eppendorf, Martinistrasse 52, 20246 Hamburg, Germany

**Keywords:** Six1, Pancreatic cancer, Epithelial-mesenchymal transition, Cancer stem cells

## Abstract

**Background:**

Epithelial-to-mesenchymal transition (EMT) and cancer stem cells (CSC) contribute to tumour progression and metastasis. Assessment of transcription factors involved in these two mechanisms can help to identify new targets for an oncological therapy. In this study, we focused on the evaluation of the transcription factor Six1 (Sine oculis 1). This protein is involved in embryologic development and its contribution to carcinogenesis has been described in several studies.

**Methods:**

Immunohistochemistry against Six1 was performed on a tissue microarray containing specimens of primary pancreatic ductal adenocarcinomas (PDAC) of 139 patients. Nuclear and cytoplasmic expression was evaluated and correlated to histopathological parameters. Expression of Six1 was inhibited transiently by siRNA in Panc1 and BxPc3 cells and stably by shRNA in Panc1 cells. Expression analysis of CDH1 and Vimentin mRNA was performed and cell motility was tested in a migration assay. Panc1 cells transfected with Six1 shRNA or scrambled shRNA were injected subcutaneously into nude mice. Tumour growth was observed for four weeks. Afterwards, tumours were stained against Six1, CD24 and CD44.

**Results:**

Six1 was overexpressed in the cytoplasm and cellular nuclei in malignant tissues (*p* < 0.0001). No correlation to histopathological parameters could be detected. Six1 down-regulation decreased pancreatic cancer cell motility in vitro. CDH1 and vimentin expression was decreased after inhibition of the expression of Six1. Pancreatic tumours with impaired expression of Six1 showed significantly delayed growth and displayed loss of the CD24^+^/CD44^+^ phenotype.

**Conclusion:**

We show that Six1 is overexpressed in human PDAC and that its inhibition results in a decreased tumour progression in vitro and in vivo. Therefore, targeting Six1 might be a novel therapeutic approach in patients with pancreatic cancer.

**Electronic supplementary material:**

The online version of this article (doi:10.1186/s12885-017-3225-5) contains supplementary material, which is available to authorized users.

## Background

Pancreatic ductal adenocarcinoma (PDAC) is a highly malignant tumour with a poor prognosis. Despite its low prevalence, it is the fourth leading cause of cancer-related death in western countries [1]. Pancreatic cancer spreads rapidly and is highly resistant to chemotherapy. These features are determined by several biological features, which are considered to be hallmarks of tumour development and dissemination [2]. Among those fundamental cornerstones, epithelial-to-mesenchymal transition (EMT) plays a crucial role in tumour progression. By adopting a more mesenchymal phenotype, cells increase motility, augment invasiveness und enhance chemoresistance [3]. EMT is strongly connected to the concept of cancer stem cells (CSC) [4]. In this model, CSC represent only a minor fraction of a tumour but are hypothesized to be crucially involved in its progression [5]. They can divide infinitely and are strongly resistant to chemotherapeutics. Therefore, they can survive chemotherapy and form recurrent disease [2]. Brabletz et al. described a model, in which migrating CSC are responsible for tumour dissemination whereas the epithelial non-CSC form is responsible for the growth of a single tumour [4]. In good accordance with this assumption, Martin et al. showed that EMT augments self-renewal capability [6].

In this study we focused on the embryologic transcription factor Six1 (Sine Oculis 1). It contributes to organogenesis by inducing proliferation, migration and survival [7–9]. In tumour biology, however, Six1 exerts pro-tumourigenic functions by regulating EMT-related mechanisms [10]. The role of Six1 in carcinogenesis has already been studied in several malignancies including breast cancer [11, 12], cervical cancer [13, 14] ovarian cancer and hepatocellular cancer [15, 16]. Recently, our group has shown that overexpression of SIX1 is an independent prognostic marker in stage I - III colorectal cancer [17]. Moreover, Li et al. [18] and Jin et al. showed an overexpression of Six1 in PDAC in their recent studies. In the study by Jin et al. Six1 was also an independent prognostic marker in pancreatic cancer [19]. Additionally, Ono et al. showed that Six1 promotes EMT by activating ZEB1 [20]. The purpose of the present study was to evaluate the impact of Six1 expression on CSC- and EMT-phenotypes in PDAC. To this end, we analysed a tissue microarray including 139 patients. Furthermore, we assessed the impact of Six1 on EMT markers and migration in vitro in Panc1 and BxPc3 cells. Finally, we investigated the impact of Six1 on tumour growth in vivo in a xenograft model.

## Methods

### Patients

The Medical Ethical Committees of the University of Bonn has approved the use of the patient tissue samples and clinic-pathological information in this study (Antragsnummer 13–091). Written informed consent was obtained from each patient prior to this study. The study cohort included 139 patients who underwent tumour resection at the University Hospital of Bonn between 1998 and 2009. The analysis was performed retrospectively and it was not possible to deduce patient identity from patient data. Cores derived from cancer tissue as well as from adjacent non-affected normal pancreatic parenchyma were analyzed.

### Immunohistochemistry

Immunohistochemical staining of human tissue microarray samples was performed as described previously [21]. Likewise, immunohistochemical staining on whole tissue specimens from xenograft samples was conducted. 2 μm sections of formalin-fixed, paraffin-embedded tumour specimens were cut and mounted on SUPERFROST® PLUS microscope slides (Menzel, Germany). After overnight incubation at 37 °C, samples were dewaxed with xylol, rehydrated in a graded series of ethanol and subjected to heat-induced antigen retrieval (Dako REAL™ Target Retrieval Solution, pH 6.00, DAKO Denmark A/S) in a pressure cooker for 15 min. Nonspecific binding was blocked using an Avidin/Biotin Blocking Kit (Vector Laboratories, Inc., Burlingame, CA, USA). After antigen retrieval, slides were placed in an automated staining machine (DAKO Automatic Stainer) and incubated with the primary antibody for 30 min. Whole tissue specimens from xenografts specimens were additionally incubated with primary antibodies against CD44 (Rabbit monoclonal, ab151037, abcam, United Kingdom) and CD24 (Rabbit monoclonal, ab17982, Abcam, United Kingdom) for 30 min. Incubation with primary antibodies was followed by the biotinylated secondary antibody (DAKO REAL™ Biotinylated Secondary Antibody Anti -Rabbit, part of the DAKO REAL™ Detection System Peroxidase/AEC, Rabbit/Mouse, Code K5003, DAKO, Denmark) for 20 min. Afterwards, endogenous peroxidase was inhibited (DAKO REAL™ Peroxidase blocking solution, DAKO, Denmark) for 5 min followed by incubation with DAKO REAL™ streptavidin peroxidase (HRP) solution (part of DAKO REAL™ Detection System Peroxidase/AEC, Rabbit/Mouse, Code K5003, DAKO, Denmark) for 20 min. Finally, the specimens were visualised with DAKO REAL™ AEC/H2O2 Substrate Solution (part of DAKO REAL™ Detection System Peroxidase/AEC, Rabbit/Mouse, Code K5003, DAKO, Denmark) and counterstained with haematoxylin. Two independent researchers (CK and TL) estimated the expression of SIX1 on a blind basis. A multi-head microscope was used and consensus was reached for each slide. The staining intensity in cytoplasm was classified as absent: 0, weak or intermediate: 1 and strong: 2. For cell nucleus staining, the percentage of positive cells was assessed: absent: 0, 0–25%: 1, 25–50%: 2, 50–75%: 3, > 75%: 4.

### Cell lines and transfection

Panc1 and BxPc3 cell lines were purchased from the American Type Culture Collection (ATCC, Manassas, VA 20108, USA). Tumour cells were maintained in RPMI-1640 (Sigma, St. Louis, MO), supplemented with 10% (*v*/v) fetal calf serum (FCS), 100 U/ml penicillin and 100 μg/ml streptomycin in a humidified atmosphere of 5% CO_2_ at 37 °C. The anti-Six1 shRNA plasmid (Mission® shRNA bacterial glycerol stock, SHCLNG-NM_005982, Sigma, USA) or an empty control vector (pLKO.1-puro, SHC001, Sigma, USA) were transfected using calcium phosphate-mediated transfection^285^ (ProFection® Mammalian, Cat. No. E1200, Promega, Germany) according to the manufacturer’s protocol. Twenty-four hours after transfection, the cells were passaged 1:15 in appropriate medium containing 1 μg/ml puromycin for puromycin selection. Transfection efficiency was determined by quantitative RT-PCR (qPCR). A transient siRNA transfection [22] was performed using Lipofectamine 2000 [23] (invitrogen, USA) according to the manufacturer’s specifications. An anti-Six1 siRNA from Sigma (Additional file 1: Table S1) and a negative control siRNA (AllStars, Qiagen, Netherlands) were purchased. Per 1200 pmol of siRNA, 30 μl of Lipofectamine 2000 were used for transfection. Afterwards, cells were incubated for 24 h and transfection efficiency was determined using quantitative RT-PCR (qPCR).

### **RNA extraction and quantitative RT-PCR** [24, 25]

Total RNA from Panc1 and BxPc3 cells was extracted with the miRNeasy Mini Kit (Qiagen, Hilden, Germany) following the manual’s instructions. RNA concentration was determined by a spectrophotometer (Nano Drop® 1000, Thermo Scientific, Germany) and reversely transcribed using the miScript Reverse Transcription Kit (Qiagen, Hilden, Germany). Five nanogram of the resulting cDNA was further subjected to qPCR (SYBR Green PCR Kit, Qiagen, Hilden, Germany) in a Roche Light Cycler™ (Roche Diagnostics GmbH, Mannheim, Germany). Ready specific primer pairs were purchased from Qiagen. Samples were normalized to GAPDH RNA and fold change of expression was calculated according to the 2^-ΔΔct^ method as previously described [26].

### Cell migration assay

The migration assay was performed using 24 well migration chambers (ThinCerts™, 8 μm pore, Greiner Bio-One, 1780 Wemmel, Belgium). Panc1 and BxPc3 cells were starved overnight. Subsequently, 20.000 cells were plated in each migration chamber in 300 μl serum-free medium. Subsequently, the migration chambers were placed on 24 well plates containing medium with 10% (*v*/v) fetal calf serum. After an incubation for 24 h, Panc1 and BxPc3 cells at the bottom of the migration chamber were stained with 4′, 6-diamidino-2-phenylindole (DAPI). 20 representative figures of each migration membrane were taken using a fluorescent microscope and the number of migrated cells of each assay was counted. All assays were performed in triplicates.

### Xenograft model

The study was approved by the regional authority for Nature, Environment and Consumer protection of the Land of North Rine-Westphalia (84–02.04.2015.A038). We used two groups each containing five mice (Athymic Nude Mouse, Crl:NU(NCr)-Foxn1^nu^, Charles River, VA, USA). 2.5 × 10^6^ cells were injected in each flank. Tumour growth and mice weight were assessed weekly for four weeks. After four weeks, the mice were euthanasized. Tumour samples were fixed in formalin and embedded in paraffin for further immunohistochemical analyses.

### Statistical analysis

The software package GraphPad Prism, version 6 (GraphPad Software, La Jolla, CA, USA) was used for all calculations. Pearson’s r test was applied to analyze the correlation between the expression of Six1 and pathological parameters. Differences in expression of Six1 in the PDAC cohort, Panc1 and BxPc3 cells, differences in migration and differences in tumour growth in vivo were assessed using the Student’s t-test. The *p* values of all statistical tests were 2-sided, and *p* ≤ 0.05 was considered to indicate a statistically significant result.

## Results

### Expression of Six1 in pancreatic ductal adenocarcinoma and its histopathological correlation

#### Patient characteristics and clinical specimens

Tissue samples from 139 patients suffering from primary pancreatic cancer were evaluated by IHC, out of these, sufficient material and data for final analysis were available in 137 cases. Of those 137 patients the median age was 66 years (36–85). 74 patients were male, 59 female. The UICC tumour stage at time of tumour resection was I in 2 cases, II in 9 cases, III in 123 cases and IV in 3 cases. 98 patients had positive lymph node metastasis (pN1), 38 patients were free of lymph node metastasis (pN0) and in 1 patient lymph node status was not known. Tumour grading was I in 1 case, II in 59 cases and III in 54 cases. In 23 cases grading could not be exactly determined. Characteristics of the cohort are shown in Table 1.

**Table 1:** Correlation of Six1 expression in cytoplasm to histopathological parameters

Parameter	Six1 expression in malignant tissue					Six1 expression in benign tissue				
	Number	No	Weak	Strong	*p*-value	Number	No	Weak	Strong	*p*-value
Total	137	50 (36,5%)	66 (48,2%)	21 (15,3%)		105	91 (86,7%)	14 (13,3%)	0	**<0,0001**
Age										
*< Median*	68 (49,6%)	26 (34,8%)	31	11	**0,844**	47 (44,8%)	38 (80,9%)	9 (19,1%)	0	**0,253**
*≥ Median*	69 (50,4%)	24 (34,8%)	35 (50,7%)	10 (14,5%)		58 (55,2%)	53 (91, 4%)	5 (8,6%)	0	
Sex^a^					**0,591**					**0,902**
*Male*	74 (56,5%)	27 (36,5%)	32 (43, 2%)	15 (20,3%)		56 (53,3%)	49 (87,5%)	(12,5%)	0	
*Female*	57 (43,5%)	19 (33,3%)	32 (56,01%)	6 (10,5%)		45 (46,7%)	39 (85,7%)	6 (14,3%)	0	
Tumor size^b^					**1,000**					**0,428**
*pT1*	2 (1,5%)	1 (50,0%)	1 (50,0%)	0		2 (1,9%)	2 (100%)	0	0	
*pT2*	9 (6,7%)	3 (33,3%)	5 (55,5%)	1 (11,1%)		8 (7,6%)	7 (87,5%)	1 (12,5%)	0	
*pT3*	121 (89,6%)	42 (34,7%)	59 (48,8%)	20 (16,5%)		90 (85,7%)	79 (87,8%)	11 (12,2%)	0	
*pT4*	3 (2,2%)	2 (66,7%)	1 (33,3%)	0		3 (2,9%)	3 (100%)	0	0	
Lymph node metastasis^c^					**0,290**					**0,959**
*N0*	38 (27,9%)	4 (10,5%)	19 (50,0%)	15 (39,5%)		31 (29,5%)	28 (90,3%)	3 (9,7%)	0	
*N1*	98 (72,1%)	34 (34,7%)	47 (48,0%)	17 (17,3%)		74 (70,5%)	63 (85,1%)	11 (14,9%)	0	
Grading					**0,950**					**0,715**
*G1*	1 (0,7%)	0	1 (100,0%)	0		1 (1,0%)	1 (100%)	0	0	
*G2*	59 (43,1%)	21 (35,6%)	28 (47,5%)	10 (16,9%)		44 (41,9%)	38 (86,4%)	6 (13,6%)	0	
*G3*	54 (39,4%)	18 (33,3%)	28 (51,2%)	8 (14,8%)		45 (42,9%)	38 (84,4%)	7 (15,6%)	0	
*Gx*	23 (14,4%)	11 (47,8%)	9 (39,1%)	3 (13,0%)		15 (14,3%)	14 (93,3%)	1 (6,7%)	0	

^a^sex was known in 131 malignant and 101 benign specimens. ^b^Tumor size was known 135 malignant and 103 benign specimens. ^c^Lymph node metastasis was only known in 136 malignant specimens

#### Expression of Six1 in human pancreatic ductal adenocarcinoma

**Fig. 1:**
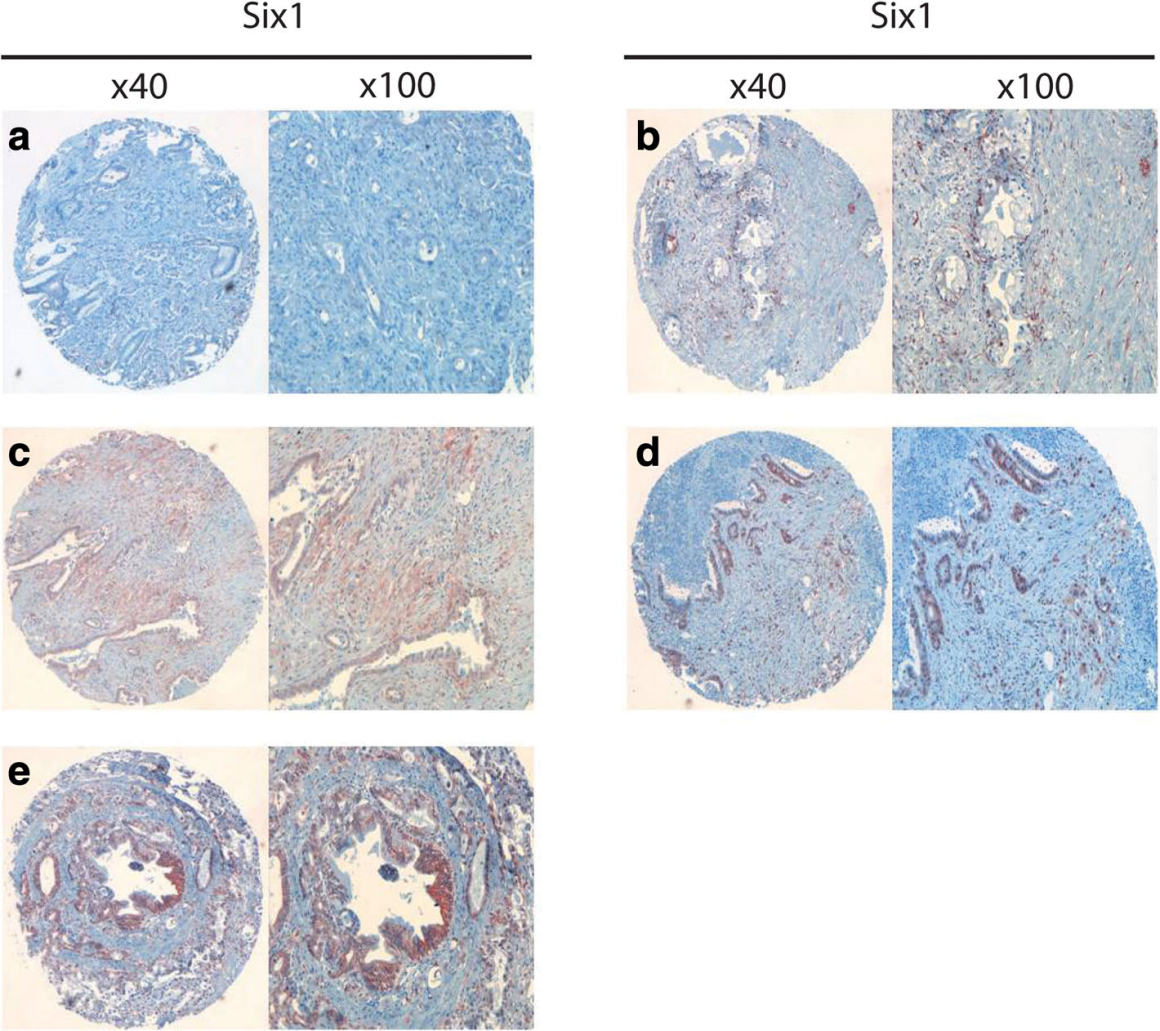
Six1-Expression in the patient cohort. Staining against Six1 was performed and Six1 expression was determined in cytoplasm and cell nucleus on a tissue microarray including human samples of patients with pancreatic cancer. **a** Negative Six1 expression in cytoplasm and nucleus **b** Negative Six1 expression in cytoplasm and positive nucleus staining **c** Weak Six1 expression in cytoplasm without nucleus staining. **d** Weak Six1 expression in cytoplasm and positive nucleus staining. **e** Strong Six1 expression in cytoplasm and negative nucleus staining. Annotations above the panel rows indicate the magnification scale of the figures: first and third row: 40× magnification. Second and fourth row: 100× magnification

We assessed Six1 expression in tissue samples derived from 139 patients with primary PDAC. In 137 out of 139 patients, cancer tissue could be evaluated and from 105 out of 139 patients, normal pancreatic parenchyma could be assessed. Evaluation was performed separately for Six1 expression in cytoplasm and nuclei, respectively (Fig. 1). 63.5 of the malignant specimens showed an expression of Six1 in the cytoplasm, whereas only 13.3% cases of benign tissue were positive for Six1 expression in the cytoplasm (*p* < 0.0001). In detail, 50 malignant tumours were negative, 66 PDAC samples showed a weak expression of Six1 and 21 tumour specimens had a high expression of Six1. In contrast, 91 specimens representing benign tissue were negative and only 14 samples displayed a weak expression of Six1 (Table 1). A positive nuclear expression of Six1 was observed in 40.8% (± 4.2) of cancer cells (0 = 81 cases; 1 = 31 cases; 2 = 17 cases, 3 = 4 cases, 4 = 4 cases). On the contrary, only 11.4% (± 3.1) of benign pancreatic tissue specimens displayed a positive expression of Six1 in the nucleus (0 = 93 cases; 1 = 10 cases, 2 = 2 cases). Further analysis between the expression of Six1 (cytoplasm and nucleus) and clinical and histopathological data revealed no significant association of those parameters: tumour stage (*p* = 1.00 and 0.40, respectively), lymph node status (0.29 and 0.48, respectively), tumour grading (0.95 and 0.19, respectively) (Table 1 and Additional file 1: Table S1).

### Inhibition of Six1 impairs cell migration in vitro

**Fig. 2:**
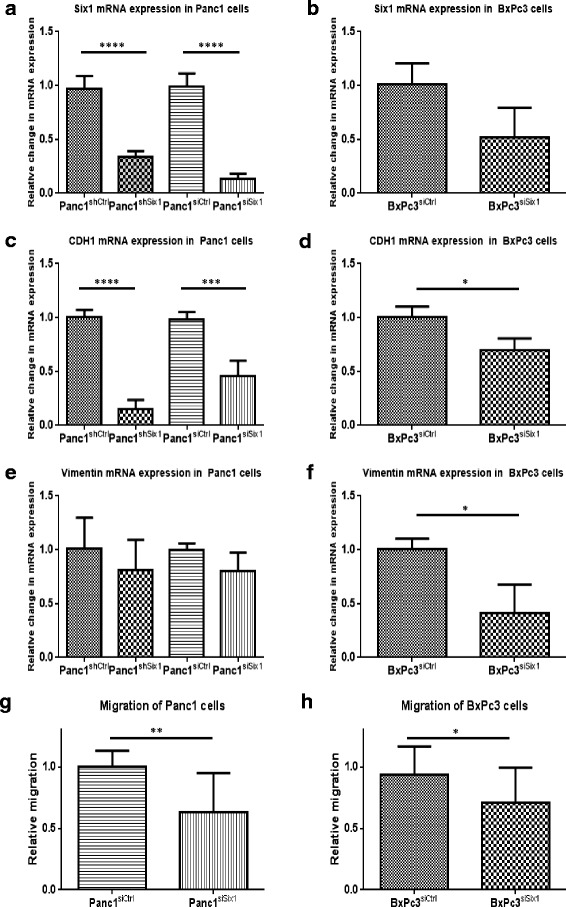
Expression of Six1, CDH1 and vimentin in Panc1 and BxPc3 cells. **a** Six1 inhibition by shRNA or siRNA decreases Six1 expression in Panc1 cells in comparison to control (scramble shRNA or siRNA). **b** Six1 inhibition siRNA decreases Six1 expression in BxPc3 cells in comparison to scramble siRNA. (C) CDH1 expression in Panc1 cells is reduced after Six1 downregulation. **d** CDH1 expression in BcPc3 is decreased after Six1 downregulation. **e** Vimentin expression in Panc1 cells is not altered by Six1 downregulation. **f** Vimentin expression in BxPc3 cells is decreased by downregulation of Six1. **g** Downregulation of Six1 impairs migration of Panc1 cells. **h** Downregulation of Six1 impairs migration of BxPc3 cells

Expression of Six1 was inhibited transiently by siRNA in Panc1 cells and BxPc3 cells. This resulted in a decreased expression of Six1 mRNA by 86% in Panc1 cells and by 48% in BxPc3 cells in comparison to controls (Fig. 2a, b). Furthermore, Six1 was stably downregulated in Panc1 cells using shRNA. This resulted in a decreased expression of Six1 by 64.5% when compared to control with scramble shRNA (Fig. 2a). Intriguingly, both approaches lead to a decreased transcription of E-Cadherin mRNA in siRNA-transfected (− 53.2%, *p* = 0.005) and shRNA-transfected (− 85.2%, *p* < 0.0001) Panc1 cells (Fig. 2c). Likewise, we observed a decreased expression of CDH1 mRNA in BxPc3 cells, when Six1 siRNA was transfected (−30%, *p* = 0.03) (Fig. 2d). Furthermore, we investigated the expression of vimentin in both cell lines. There was a slight, but not significant decrease in siRNA-transfected and shRNA- Panc1 cells (Fig. 2e). However, BxPC3 transfected with siRNA against Six1 showed a significantly declined expression of vimentin by 58.6% (*p* = 0.04) (Fig. 2f).

To assess the impact of Six1 inhibition on cell motility, we performed migration assays with Panc1 and BxPc3 cells, which had been transfected with siRNA against Six1 or control. Inhibition of Six1 reduced migration in Panc1 cells by 37% (*p* = 0.008) and in BxPc3 cells by 24.2% (*p* = 0.031) (Fig. 2g, h, Additional file 2: Fig. S1).

### Effects of Six1 down regulation in Panc1 cells in vivo

**Fig. 3:**
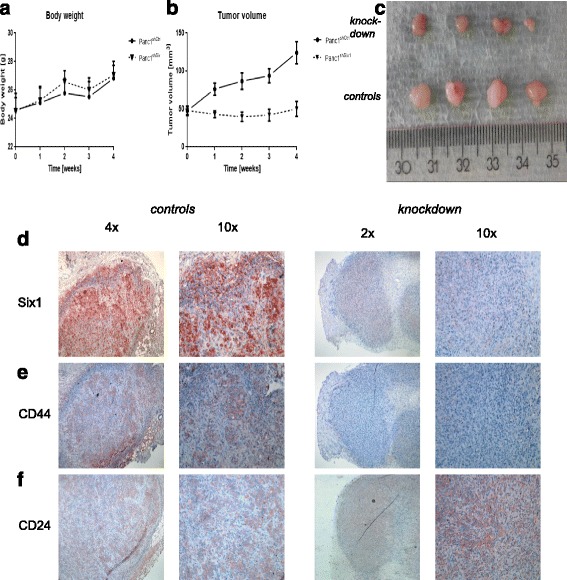
Six1 downregulation results in a growth arrest of Panc1 cells in a xenograft model. **a** Body weight curve of mice. Straight line: Panc-1 tumours with scramble shRNA (Panc1^shCtl^). Dashed line: Panc-1 tumours with Six1-shRNA (Panc1^shSix1^). No difference in body weight in both groups. **b** Tumour growth curve of Panc-1 tumours. Straight line: Panc-1 tumours with scramble shRNA (Panc1^shCtl^). Dashed line: Panc-1 tumours with Six1-shRNA (Panc1^shSix1^). **c** Tumour volume of Panc-1 tumour after resection from xenograft models. Upper panel: Panc-1 tumours with Six1-shRNA (Panc1^shSix1^). Lower panel: Panc-1 tumours with scramble shRNA (Panc1^shCtl^). **d** Representative figures for expression of Six1 in Panc-1 tumours with scramble shRNA (*left panel*) and tumours with Six1-shRNA (*right panel*). **e**,**f** Representative figures for expression of CD44 **e** and CD24 **f** in Panc-1 tumours with scramble shRNA (*left panel*) and tumours with Six1-shRNA (*right panel*)

We assessed the effect of Six1 inhibition in Panc1 cells in vivo in a xenograft model. For this analysis, shRNA-transfected Panc1 cells were used, showing a stably decreased expression of Six1. After injection of tumour cells into nude mice (2.5 × 10^6^ cells/mouse), body weight and tumour growth were observed for four weeks (Fig. 3a–c). The body weight did not show any difference between these two groups during the observation time. In the beginning, the average tumour volume was 47.65 mm^3^ (±19.34) in the control group (Panc1^shctrl^) and 47.00 mm^3^ (±15.90) in the group with Six1-shRNA (Panc1^shSix1^). After two weeks, the tumour volume had increased to 86.78 mm^3^ (±33.93) in the control group and had declined to 39.84 mm^3^ (±18.54) in Panc1^shSix1^group (*p* = 0.0018). At time of euthanasia, the average tumour volume was 124.13 mm^3^ (±46.59) in the Panc1^shctrl^ group and 50.22 mm^3^ (±29.76) in Panc1^shSix1^ (*p* = 0.0008). After euthanasia, tumour samples of both groups were immunostained against Six1. As expected, tumours from the Panc1^shctrl^ group showed a higher expression of Six1 than tumours from the Panc1^shSix1^ group (Fig. 3d). Interestingly, in the tumour specimens of the Panc1^shctrl^ group, we observed an increased expression of Six1 at the invasive edge where EMT plays an important role for tumour invasion. Moreover, we evaluated the expression of CD44 and CD24 in those murine tumour samples to assess the co-expression of EMT markers and surrogate markers associated with a CSC phenotype [27]. Four out of five control tumours were CD44^+^/CD24^+^ whereas all Six1-downregulated tumours lost that phenotype and were CD44^−^/CD24^+^ (Fig. 3d, e, and f and Additional file 3: Table S2).

## Discussion

Pancreatic cancer (PDAC) is one of the most aggressive types of tumours. For the last decade, its tumour biology has been more and more elucidated, revealing the important role of EMT in tumour progression. Therefore, in this study, we focused on Six1, which originally has been described as an EMT regulator under physiological conditions in numerous types of tissue. However, its role in carcinogenesis has also become more evident recently [11, 12, 17]. In PDAC, two studies have investigated the role of Six1 so far [18, 19]. They demonstrated that overexpression of Six1 is associated with tumour stage, lymph node status and grading. Furthermore, Li et al. [18] showed that increased expression of Six1 is an independent prognostic marker for survival in pancreatic cancer. Our cohort consisted of 139 patients suffering from primary PDAC who were operated on at the university hospital in Bonn between 1998 and 2009. To our knowledge, this is the largest cohort of patients with PDAC in which the expression of Six1 has been investigated to date. In 137 malignant and 105 benign samples, Six1 expression was assessed. In accordance with the two previous studies, we observed an overexpression of Six1 in cancer cells compared to healthy tissue. In contrast, we did not find a significant correlation between the expression of Six1 and any clinical or histopathological data. These controversial observations may be explained by the different clinical characteristics of our cohort in comparison to the previous studies: in our analysis, almost all tumours were diagnosed as stage pT3, grading G2 or G3 and lymph node status pN1. On the contrary, the population of Jin et al. was more heterogenous and tumours were in a less advanced stage. Taking into account the very homogenous characteristics of our cohort, statistical analysis would require a much higher number of participants to find a significant correlation.

EMT has been described as one of the hallmarks of cancer [2]. It increases motility and invasiveness. In line with this hallmark, we demonstrated that decreased expression of Six1 results in an impaired motility of Panc1 and BxPc3 cells. Several proteins were proposed as surrogate markers of EMT. CDH1 is a protein involved in cell-cell-contacts, thereby often used as marker for epithelial character [28]. Vimentin is an intermediate filament used as a surrogate for mesenchymal differentiation [29]. Under the assumption that Six1 induces a more mesenchymal phenotype, one would conjecture that Six1 down-regulation results in an increased expression of CDH1 and a decreased expression of vimentin. Intriguingly, in our study we were able to show by several independent experiments that decreased expression of Six1 induces a declined transcription of CDH1 in Panc1 and in BxPc3 cells. Moreover, reduced expression of Six1 slightly affected the expression of vimentin in Panc1, but showed a decreased regulation of Vimentin in BxPc3 cells. To some extent, these data are diametrically opposed to the assumed results. It is unlikely that the decreased expression of CDH1 is an arbitrary result of our transfection method or of the used siRNA. We performed our experiments in two different cell lines. Moreover, the expression of Six1 was reduced significantly by siRNA and by shRNA in comparison to scramble shRNA or control siRNA, respectively. In the light of this, our results underscore that both, CDH1 and Vimentin, are rather surrogate markers for EMT. There exists no definite and unique EMT marker. Cells undergoing EMT exhibit rather a dynamic phenotype, where epithelial and mesenchymal features occur in the same time. This is part of the EMT-MET (mesenchymal-to-epithelial transition) axis which regulates the phenotype of a pro-invasive (mesenchymal) state and an epithelial phenotype. Both types of differentiation are important for tumour spread: the mesenchymal state is important for tumour invasion whereas the epithelial state is required for the colonisation and formation of metastasis in distant organs. Such biphasic effects have also been observed for other EMT-related transcription factors, such as TWIST1 [30]. This transcription factor can induce the expression of miR-424, potentially facilitating earlier, but repressing later stages of metastasis by regulating an EMT-MET axis [30]. It remains speculative but our data may indicate that inhibition of Six1 may affect the EMT-MET axis by regulating both mesenchymal and epithelial genes. However, a relative preponderance of mesenchymal processes versus epithelial processes may shift the balance towards a pro-migratory phenotype, which may explain the results of our migration assays.

Finally, we investigated the impact of Six1 in Panc1 cells in a xenograft model. In this experiment we could observe that tumour growth was impaired significantly when the expression of Six1 was decreased in stable transfected clones by shRNA. These data are in good accordance with the assumption that the inhibition of EMT-related transcription factors results in a diminished tumour growth [31]. To further elucidate those findings, we evaluated the expression of cancer stem cell (CSC) markers in the murine xenograft tumours. Ford et al. have described that Six1 increases the population of CSCs in breast cancer [32]. Conclusively, we hypothesized that decreased expression of Six1 would also result in a reduced number of tumour cells with a CSC-phenotype. We therefore analysed the expression of CD24 and CD44 since Li et al. [27] had identified CD24^+^/CD44^+^/ESA^+^ cells as pancreatic cancer stem cells. In our experiment, control tumours displayed a significantly stronger expression CD24^+^/CD44^+^ cells than tumours with down-regulation of Six1. The latter were characterised by cells with a CD24^+^/CD44^−^ phenotype, which represents cells with less CSC features. These findings may suggest that decreased expression of Six1 impairs fundamental CSC functions which also results in a less aggressive and less invasive phenotype. Although this result is in good accordance with biological hypotheses and findings in breast cancer, further studies would certainly be warranted to better characterize the effects of Six1 on CSC induction in PDAC in vitro and in vivo.

## Conclusion

In conclusion, in the largest cohort of patients studied so far, we confirm the results of previous reports that Six1 is overexpressed in PDAC. Furthermore, we show that inhibition of Six1 leads to decreased cell motility in Panc1 and BxPc3 cells. These results are in good accordance with the hypothesis that Six1 induces EMT. Interestingly, CDH1 mRNA expression was also decreased by impaired expression of Six1 which deserves further investigation in following studies and may reflect a biphasic effect of Six1 on the EMT-MET axis. Moreover, our data show that stable inhibition of Six1 decreases tumour growth in a xenograft model. This is associated with a decreased expression of CSC-markers in the tumour tissue. Overall, our results provide further evidence that Six1 co-promotes tumour progression in pancreatic cancer. Therefore, targeting Six1 might be a novel promising therapeutic approach in patients with pancreatic cancer.

## Additional files

Additional file 1: Table 1. Six1 expression in the cell nucleus of malignant and benign tissue and its correlation to clinical and histopathological parameters. *Only 135 malignant and 103 benign specimens could be included. **Only 136 malignant specimens could be evaluated. (DOCX 14 kb)

Additional file 2: Fig. S1. Sequence of siRNA against Six1R1. (PPTX 3958 kb)

Additional file 3: Table 2. Six1, CD44, CD24 and tumor volumes in each mice. (DOCX 11 kb)

## Abbreviations

CSC: Cancer stem cells
EMT: Epithelial-mesenchymal transition
PDAC: Pancreatic ductal adenocarcinoma
