# Supplementary material for: Inhibition of Six1 affects tumour invasion and the expression of cancer stem cell markers in pancreatic cancer
Source: BMC Cancer. 2017 Apr 7;17:249. doi: 10.1186/s12885-017-3225-5 (PMC5383957; doi:10.1186/s12885-017-3225-5)
Supplement: Supplementary file 1 — Six1 expression in the cell nucleus of malignant and benign tissue and its correlation to clinical and histopathological parameters. *Only 135 malignant and 103 benign specimens could be included. **Only 136 malignant specimens could be evaluated. (DOCX 14 kb) [file 12885_2017_3225_MOESM1_ESM.docx]

| **Parameter** | **Number** | **0 %** | **0 – 25 %** | **25 – 50 %** | **50 – 75 %** | **≥ 75 %** | **p-value** |
| --- | --- | --- | --- | --- | --- | --- | --- |
| **Total**  **(Malignant)** | 137 | 81  (59,1 %) | 31  (22,6 %) | 17  (12,4 %) | 4  (2,9 %) | 4  (2,9 %) |  |
| **Total**  **(Benign)** | 105 | 93  (88,6 %) | 10  (9,5 %) | 2 | 0 | 0 |  |
| **Age** |  |  |  |  |  |  |  |
| *< Median* | 68  (49,6 %) | 37  (54,4 %) | 14  (20,6 %) | 13  (19,1 %) | 1  (1,5 %) | 3  (4,4 %) | **0,186** |
| *≥ Median* | 69  (50,4 %) | 44  (63,8 %) | 17  (24,6 %) | 4  (5,8 %) | 3  (4,3 %) | 1  (1,4 %) |  |
| **Sex** |  |  |  |  |  |  | **0,918** |
| *Male* | 74  (56,5 %) | 45  (60,8 %) | 16  (21,6 %) | 11  (14,9 %) | 2  (2,7 %) | 0 |  |
| *Female* | 59  (43,5 %) | 35  (59,3 %) | 14  (23,7 %) | 6  (10,2 %) | 1  (1,7 %) | 1  (1,7 %) |  |
| **Tumor size*** |  |  |  |  |  |  | **0,395** |
| *pT1* | 2  (1,5 %) | 2  (100 %) | 0 | 0 | 0 | 0 |  |
| *pT2* | 9  (6,7 %) | 7  (77,8 %) | 0 | 2  (22,2 %) | 0 | 0 |  |
| *pT3* | 121  (89,6 %) | 69  (57,0 %) | 30  (24,8 %) | 14  (11,6 %) | 4  (3,3 %) | 4 (3,3 %) |  |
| *pT4* | 3  (2,2 %) | 2  (66,7 %) | 1  (33,3 %) | 0 | 0 | 0 |  |
| **Lymph node metastasis**** |  |  |  |  |  |  | **0,482** |
| *N0* | 38  (27,9 %) | 28  (73,7 %) | 2  (5,3 %) | 5  (13,2 %) | 2  (5,3 %) | 1  (2,6 %) |  |
| *N1* | 98  (72,1 %) | 52  (53,1 %) | 29  (29,6 %) | 12  (12,2 %) | 2  (2,0 %) | 3  (3,1 %) |  |
| **Grading** |  |  |  |  |  |  | **0,190** |
| *G1* | 1  (0,7 %) | 0 | 0 | 0 | 1  (100,0 %) | 0 |  |
| *G2* | 59  (43,1 %) | 33  (55,9 %) | 17  (28,8 %) | 6  (10,2 %) | 0 | 3  (5,1 %) |  |
| *G3* | 54  (39,4 %) | 33  (61,1 %) | 12  (22,2 %) | 8  (14,8 %) | 1  (1,9 %) | 0 |  |
| *Gx* | 23  (14,4 %) | 15  (65,2 %) | 2  (8,7 %) | 3  (13,0 %) | 2  (8,7 %) | 1  (4,3 %) |  |

**Supplementary table 1. Six1 expression in the cell nucleus of malignant and benign tissue and its correlation to clinical and histopathological parameters.** *Only 135 malignant and 103 benign specimens could be included. **Only 136 malignant specimens could be evaluated.
