# Supplementary material for: Inhibition of Six1 affects tumour invasion and the expression of cancer stem cell markers in pancreatic cancer
Source: BMC Cancer. 2017 Apr 7;17:249. doi: 10.1186/s12885-017-3225-5 (PMC5383957; doi:10.1186/s12885-017-3225-5)
Supplement: Supplementary file 2 — Sequence of siRNA against Six1R1. (PPTX 3958 kb) [file 12885_2017_3225_MOESM2_ESM.pptx]

## Slide 1
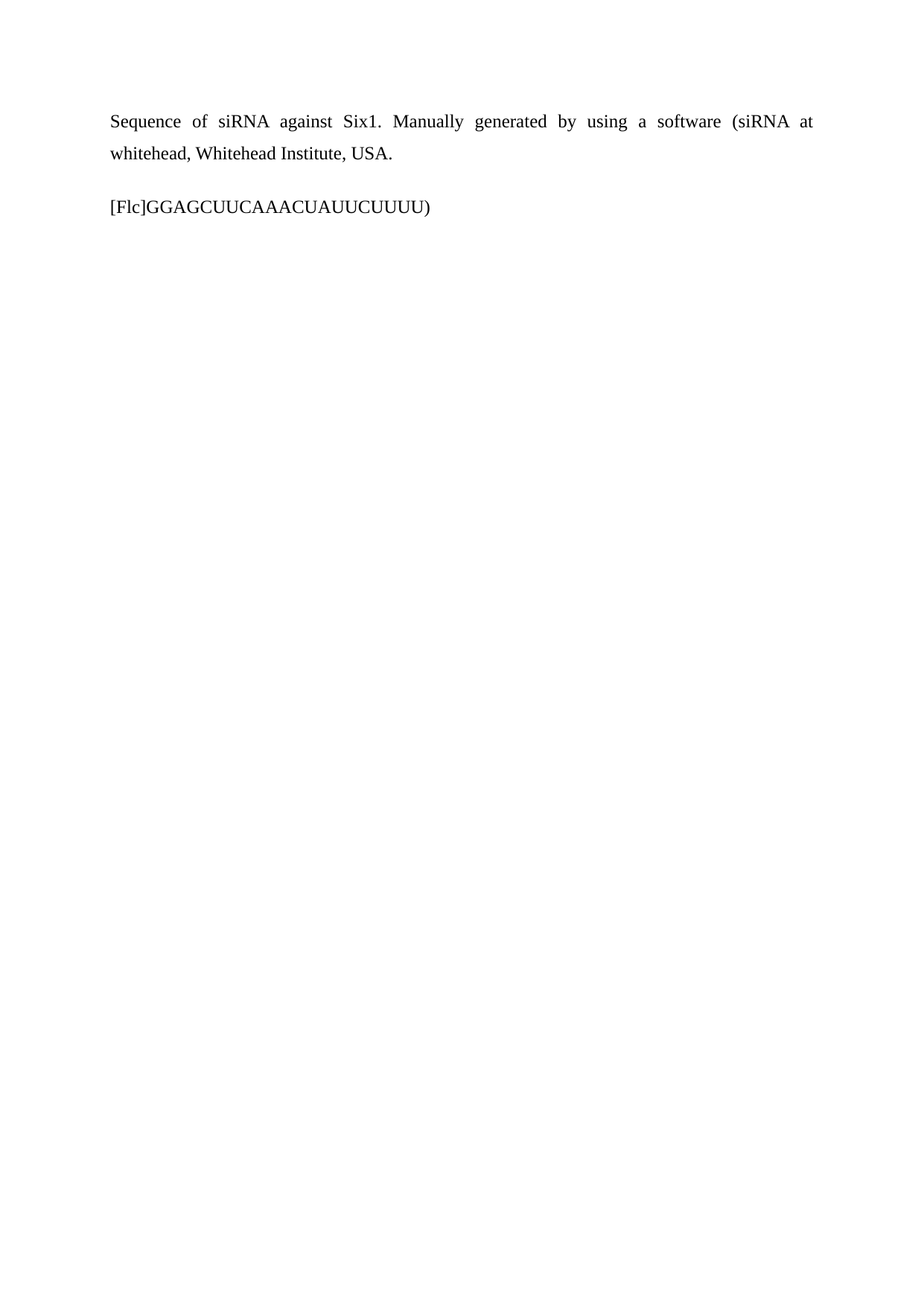

Sequence of siRNA against Six1. Manually generated by using a software (siRNA at whitehead, Whitehead Institute, USA.
[Flc]GGAGCUUCAAACUAUUCUUUU)
