# Supplementary material for: Inhibition of Six1 affects tumour invasion and the expression of cancer stem cell markers in pancreatic cancer
Source: BMC Cancer. 2017 Apr 7;17:249. doi: 10.1186/s12885-017-3225-5 (PMC5383957; doi:10.1186/s12885-017-3225-5)
Supplement: Supplementary file 3 — Six1, CD44, CD24 and tumor volumes in each mice. (DOCX 11 kb) [file 12885_2017_3225_MOESM3_ESM.docx]

| Mice | Clone | Six1 | CD44 | CD24 | Tumor volume[mm^3^] |
| --- | --- | --- | --- | --- | --- |
| 1 | Control | Positive | Positive | Positive | 171,03 |
| 2 | Control | Positive | Positive | Positive | 200,46 |
| 3 | Control | Positive | Positive | Positive | 355,19 |
| 4 | Control | Negative | Negative | Positive | 323,56 |
| 5 | Control | Positive | Positive | Positive | 191,10 |
| 6 | Knockdown | Negative | Negative | Positive | 131,91 |
| 7 | Knockdown | Negative | Negative | Positive | 63,75 |
| 8 | Knockdown | Negative | Negative | Positive | 133,29 |
| 9 | Knockdown | Negative | Negative | Positive | 84,45 |
| 10 | Knockdown | Negative | Negative | Positive | 37,56 |

**Supplementary appendix 2.** Six1, CD44, CD24 and tumor volumes in each mice.
